# Anaesthetic Challenges in a Case of Oral Carcinoma With Anticipated Difficult Airway Posted for Tumour Excision and Reconstruction Surgery

**DOI:** 10.7759/cureus.34599

**Published:** 2023-02-03

**Authors:** Sambit Dash, Nikhil Bhalerao, Aditi Gaurkar, Shiras P, Aruna Chandak

**Affiliations:** 1 Department of Anesthesiology, Jawaharlal Nehru Medical College, Acharya Vinoba Bhave Rural Hospital, Datta Meghe Institute of Higher Education & Research, Wardha, IND

**Keywords:** awake intubation, fibreoptic bronchoscope, predicted difficult airway, reconstruction surgery, anaesthesia

## Abstract

Mandibular surgery, edentulous jaw, denture wear, and ageing are all risk factors for persistent mandibular ridge resorption and weakening. The tongue occludes the upper airway due to the mandible's edentulous condition. All of these factors contribute to the difficulties in regulating the airway. An adequate preoperative review assisted in classifying this index patient as having a high risk of difficult airway management, and appropriate actions were made to facilitate effective airway care. A 60-year-old male presented to casualty with a complaint of squamous cell carcinoma of the right buccal mucosa and was posted for wide local excision of the tumour, segmental mandibulectomy, bilateral modified radical neck dissection, and reconstruction with a fibular free flap. He had a restricted mouth opening and a heavy jaw, with Mallampati grade 4 and had an anticipated difficult airway. Hence, awake endotracheal intubation was done by flexible fibreoptic bronchoscope following airway blocks and an 8.0 mm cuffed flexometallic armoured tube was secured at 28 cm at the angle of the nose. Bilateral modified radical neck dissection and wide local excision of the tumour were done followed by mandibulectomy and its reconstruction by fibular free flap and anastomosis was performed. Tracheostomy was performed and the patient was shifted to the intensive care unit and kept knocked out with injection vecuronium and injection midazolam infusion. The patient was gradually weaned off the ventilator the following day and discharged on postoperative day 12 with minimal postoperative complications. A thorough pre-anaesthetic plan, simple and skilled anaesthetic management strategy, and well-organized teamwork aided in the effective anaesthetic care of this challenging airway patient.

## Introduction

Oral cancer is the sixth most prevalent cancer worldwide [1]. It is the most prevalent cancer among males in India [2] and is associated with tobacco and gutka use. Even if the human papillomavirus has lately been linked to the development of oral cancer, other more widespread causes in India must not be neglected. Surgery is the first-line therapy for oral cancer [1]. Due to the reduced mouth opening and decreased interincisor space, airway difficulty is a primary anaesthetic issue during surgery. Patients with oral cancer who undergo radiation as their primary treatment are more likely to experience limited neck motion and extension issues, as well as restricted mouth opening. Taking these considerations into account, the competence and judgement of anaesthesiologists will lower morbidity and death without question.

To heal an existing lesion, the words free flap, island flap, free autologous tissue transfer, and microvascular free tissue transfer are used to describe the transplantation of tissue from one region of the body to another. The word "free" refers to the removal of tissue from the original place (donor) to another site (receiver) where circulation is established by arterial and venous anastomosis. As free flaps, it is possible to transplant skin and subcutaneous tissue, muscle, nerve, bone, cartilage (or any combination thereof), lymph nodes, and intestine segments. In the 1960s, Goldwyn and Krizek developed microvascular anastomosis enabling free tissue transplantation in animal models [3,4]. Daniel and Taylor conducted the first documented successful free flap in a clinical instance in 1973, which heralded the discovery of an iliofemoral island flap and its subsequent long-distance transfer [5].

In recent years, the use of free or pedicle flaps for defect restoration has gained popularity in head and neck reconstructive surgery, which has seen major advancements. Ariyan introduced the pectoralis major myocutaneous flap supplied by the pectoral branch of the thoracoacromial artery in 1979, and it is presently utilized in several centres for head and neck reconstruction [3]. Concerns about the dependability of the flap for some flaws led to the widespread use of free flaps for big head and neck malformations. Consequently, free flaps need a higher level of microvascular surgical competence and a longer operating time. However, two surgical teams, one at the donor location and the other at the recipient site, might shorten the duration of the surgery in modern times. Increased operation duration, on the other hand, increases the patient's exposure to anaesthetics.

## Case presentation

**Figure 1:**
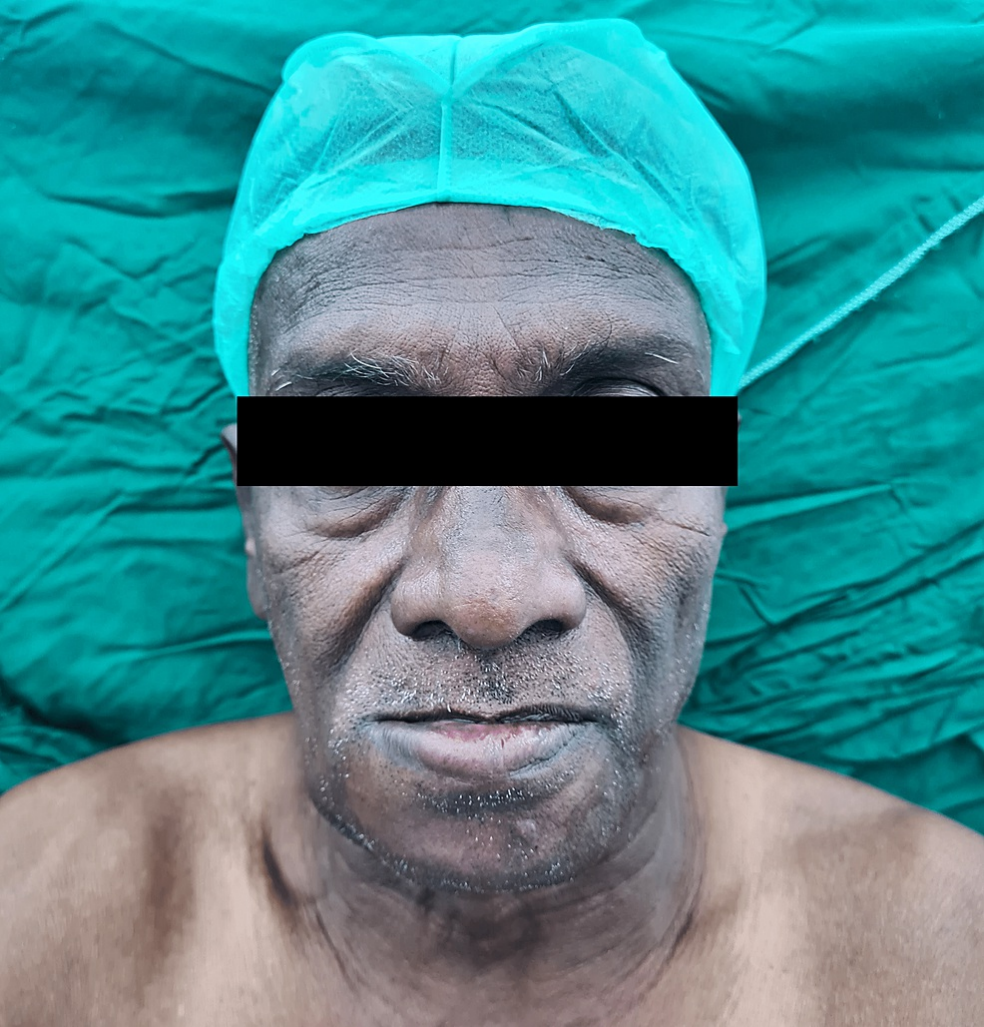
Image of the patient with biopsy-proven squamous cell carcinoma of the right gingivobuccal sulcus.

**Figure 2:**
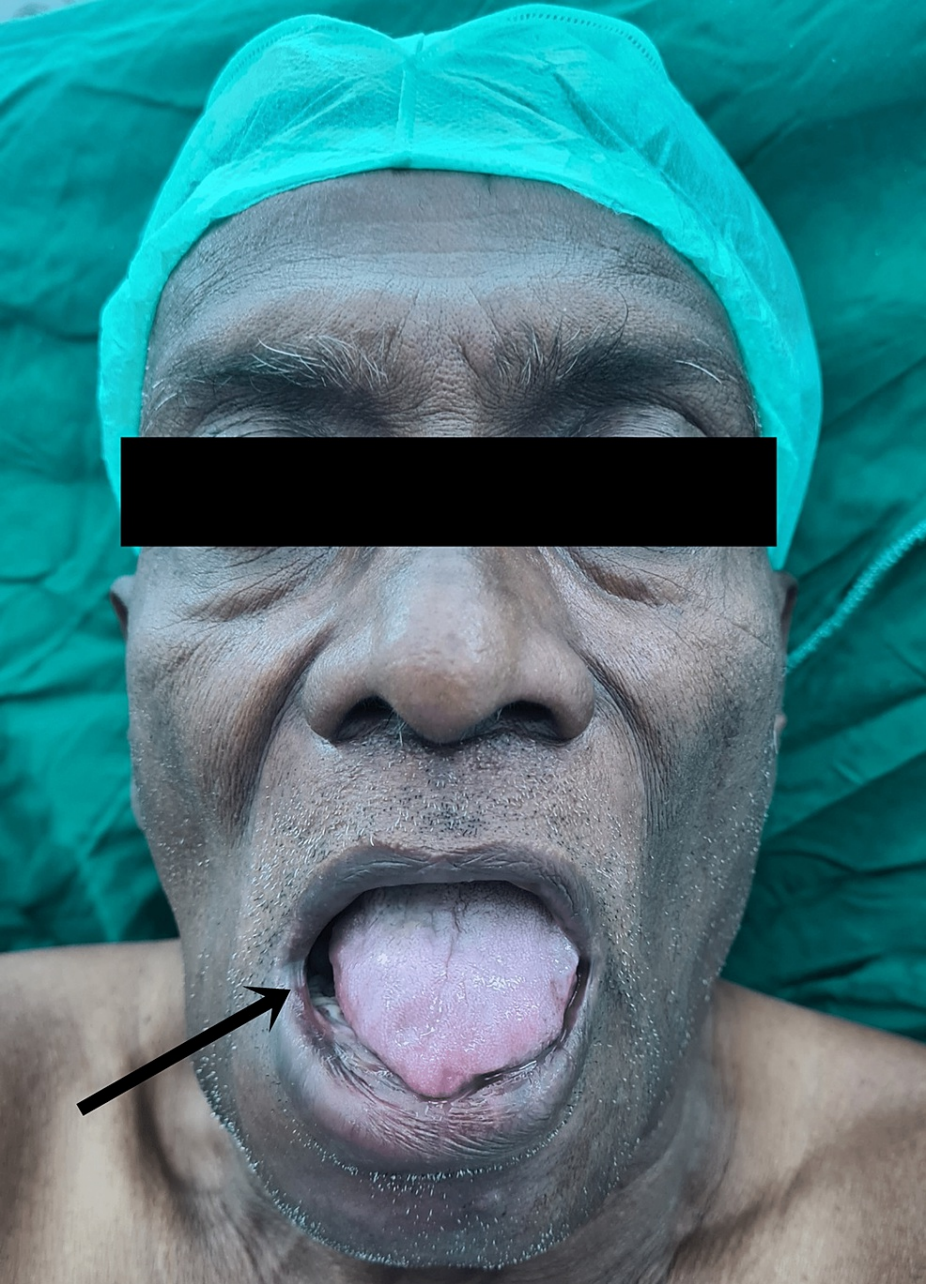
The Mallampati classification of the patient was found to be class IV, indicative of difficult intubation and airway management.

A 60-year-old male presented to the casualty with complaints of pain in the floor of the right side of the mouth for one month, which was associated with swelling and tenderness for the last seven days. After the initial assessment, the patient was referred to the oncological surgery department for further assessment. On detailed questioning, the patient gave a history of chronic tobacco consumption and chronic alcohol intake for the last 30 years. A biopsy was taken from the lesion and was sent for histopathology and routine investigations were done. The sample came positive for squamous cell carcinoma. The patient was planned for wide local excision of the tumour, segmental mandibulectomy, bilateral modified radical neck dissection, and reconstruction with fibular free flap (Figure 1). During the pre-anaesthetic examination, the patient was found to be hypertensive with a blood pressure of 168/102 mmHg. A detailed cardiac examination was done. ECG showed features of left axis deviation, and two-dimensional echocardiography revealed left ventricular hypertrophy with an ejection fraction of 55% and no other valvular anomalies. On airway examination, the mouth opening was two fingers and Mallampati classification was class IV, indicating only visualization of only hard palate (Figure 2).

**Figure 3:**
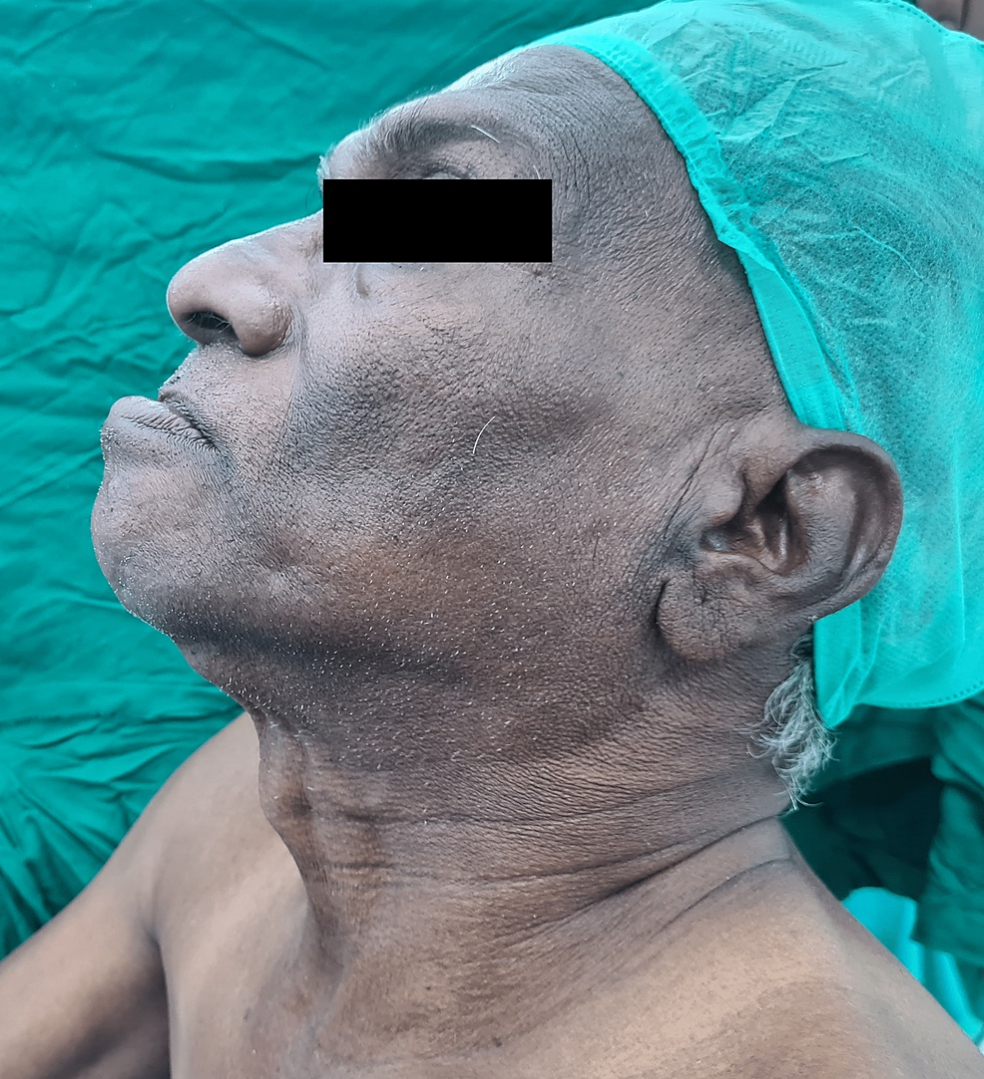
The neck extension of the patient was found to be adequate.

**Figure 4:**
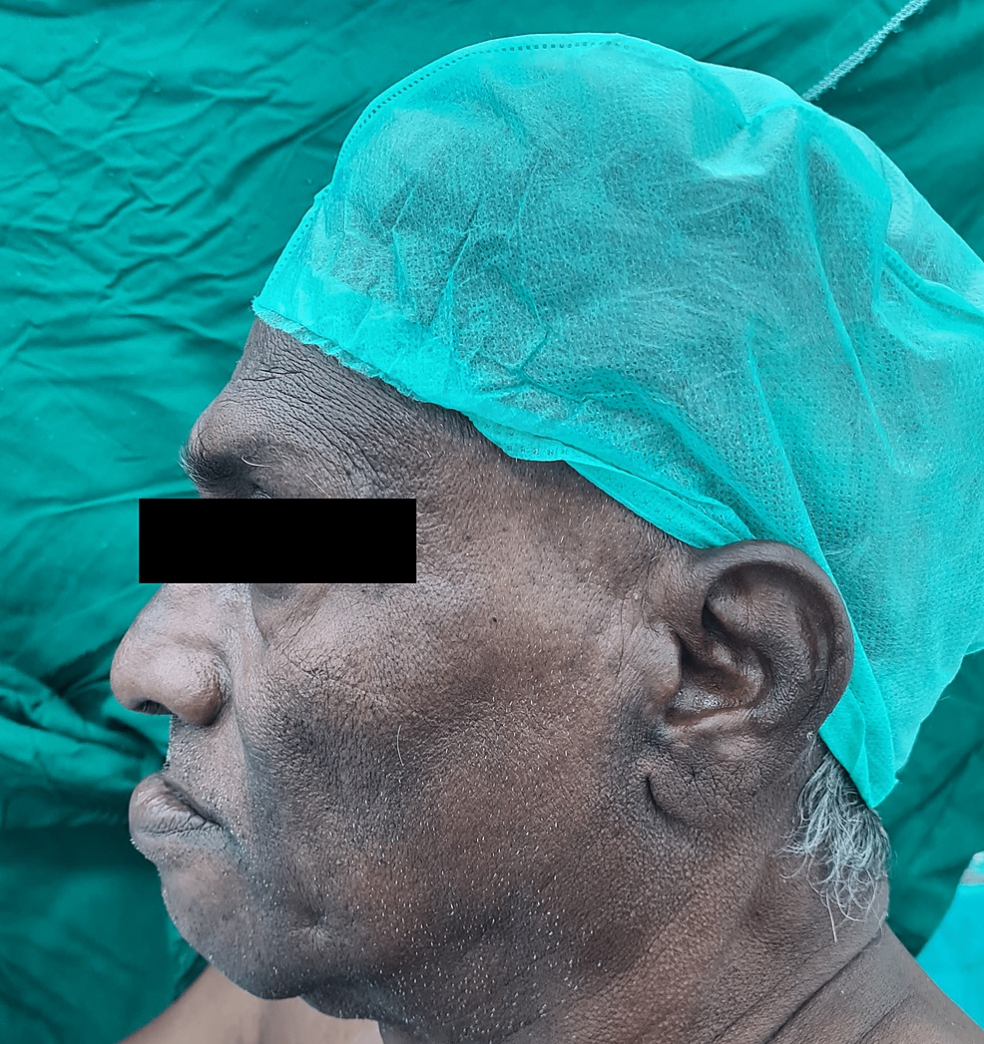
Mandibular protrusion test done was found to be class A, which indicates the lower incisor can be protruded anterior to the upper incisors.

The neck movement was adequate while the temporomandibular joint movement was restricted due to an oral lesion, which was associated with pain (Figure 3). The mandibular protrusion test done was found to be class A, which indicates the lower incisor can be protruded anterior to the upper incisors (Figure 4). All lab investigations including complete blood count (CBC), kidney function test (KFT), and liver function test (LFT) were found to be within normal limits. A fibreoptic bronchoscope with awake intubation was planned. The Enhanced Recovery After Surgery (ERAS) protocol was followed for the patient. Preoperative counselling was provided, and adequate preoperative nutrition was maintained. The patient was kept nil by mouth for eight hours prior to surgery, with adequate IV fluid maintenance. After obtaining written and verbal consent for high-risk treatment from the patient and his relatives, the patient was transferred to the operation table. On both hands, two 16 G peripheral intravenous catheters were secured. Prior to starting of procedure, in the preoperative room, the patient was nebulized with 4% lignocaine, which was delivered in conjugation with oxygen for up to 30 minutes in a noninvasive way to topicalize the airway all the way down to the trachea. It is well tolerated and is a useful technique to topicalize the whole airway. It also allows the topicalization of patients with limited mouth opening, similar to our case.

**Figure 5:**
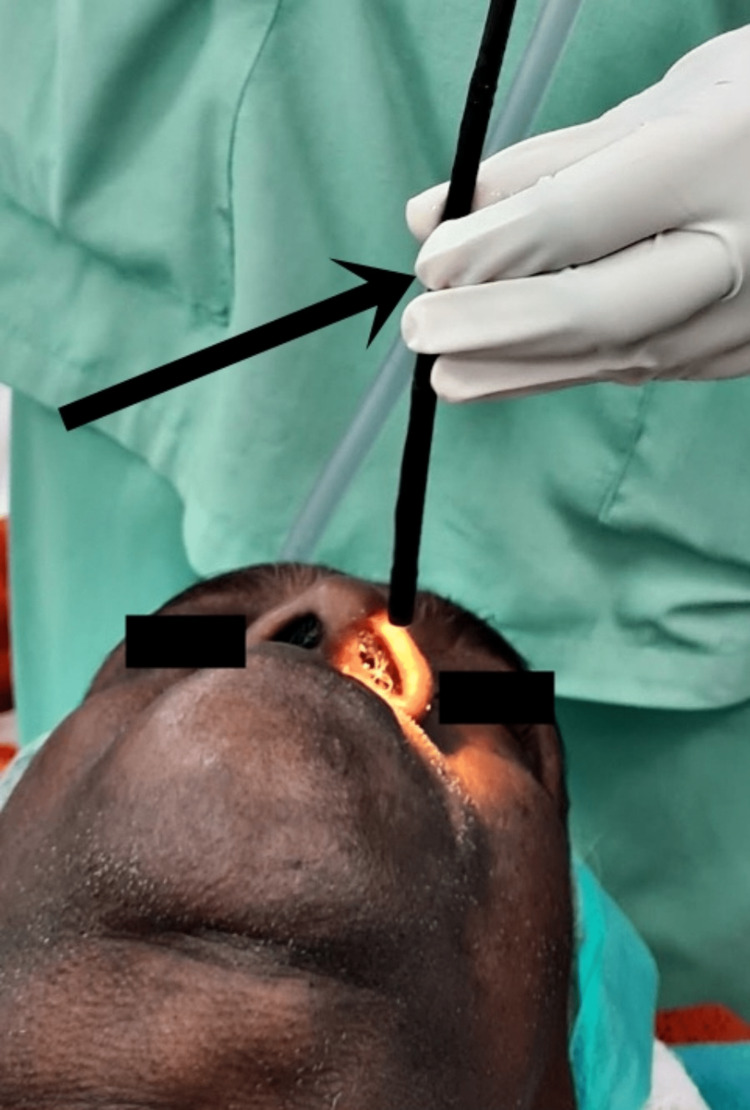
Image showing the bronchoscope being introduced through the left nostril indicated by the arrow.

Lignocaine 10% spray was applied to the base of the posterior tonsillar pillars (palatopharyngeal arch) to block the glossopharyngeal nerve. A superior laryngeal nerve block was performed by injection of lignocaine 2% 2 ml on either side of the greater cornu of the hyoid bone to block both the external and internal branches of the superior laryngeal nerve. The recurrent laryngeal nerve was blocked by a transtracheal approach, and 2 ml of lignocaine 4% injection was injected with a 22 gauge inserted perpendicular to the skin with the aim to penetrate the cricothyroid membrane (above the cricoid cartilage) with the continuous aspiration of the syringe, as the appearance of bubbles indicated that the needle tip is now in the trachea. Rapid injection (and then removal of the needle) of 2 ml of 4% lidocaine resulted in coughing, which helped to disperse the local anaesthetic and block the recurrent laryngeal nerve. After the airway was ready with blocks, glycopyrrolate 0.2 mg injection, midazolam 1 mg injection, and fentanyl 50 mcg injection were given intravenously. An 8.0 mm cuffed armoured flexometallic tube was taken and railroaded on the bronchoscope. The bronchoscope was inserted from the left nostril (Figure 5). After visualization of the vocal cords and the tracheal rings, the bronchoscope was guided to the level of carina under direct visualization followed by sliding of the flexometallic tube over the bronchoscope.

**Figure 6:**
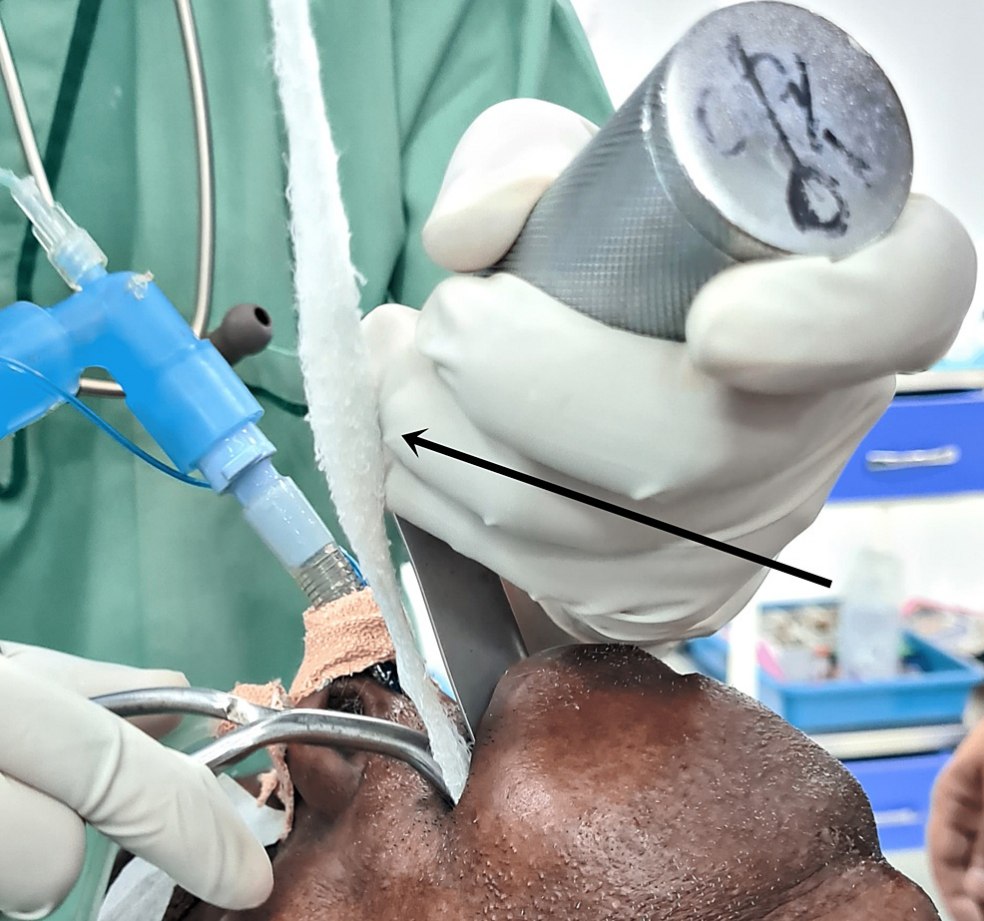
Image showing insertion of throat pack to prevent microaspiration.

**Figure 7:**
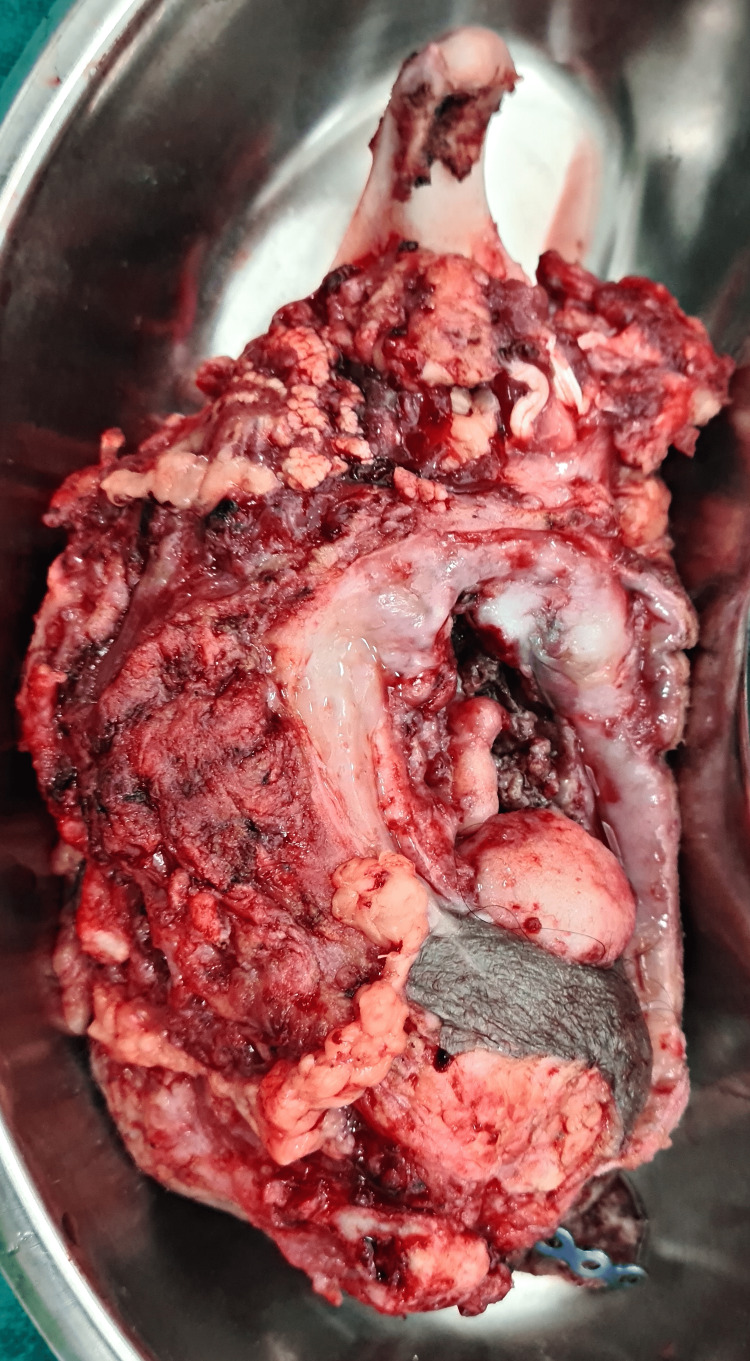
Image of the specimen post mandibulectomy.

**Figure 8:**
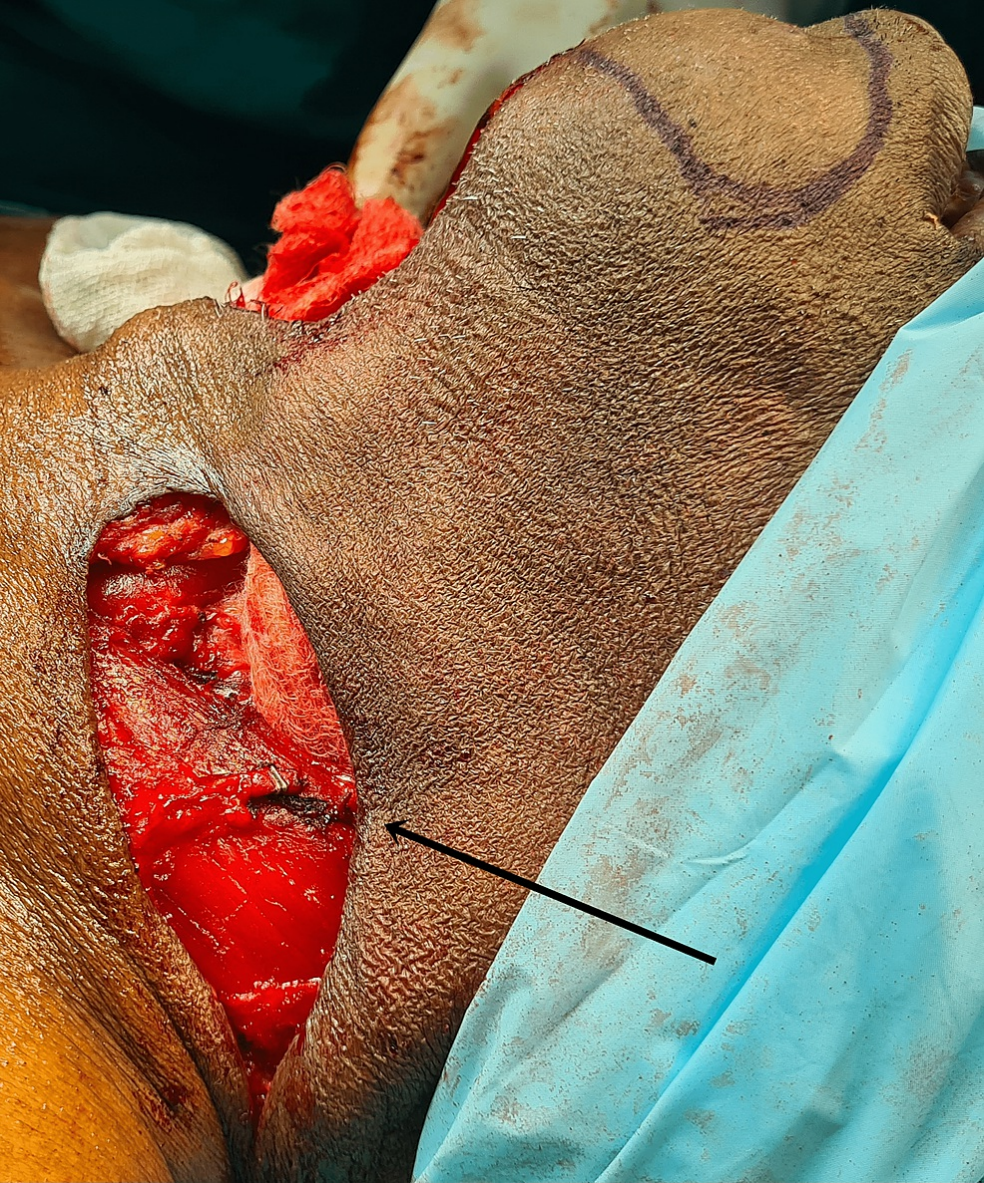
Image showing bilateral radical neck dissection done to remove the lymph nodes, which was then sent for histopathological studies.

**Figure 9:**
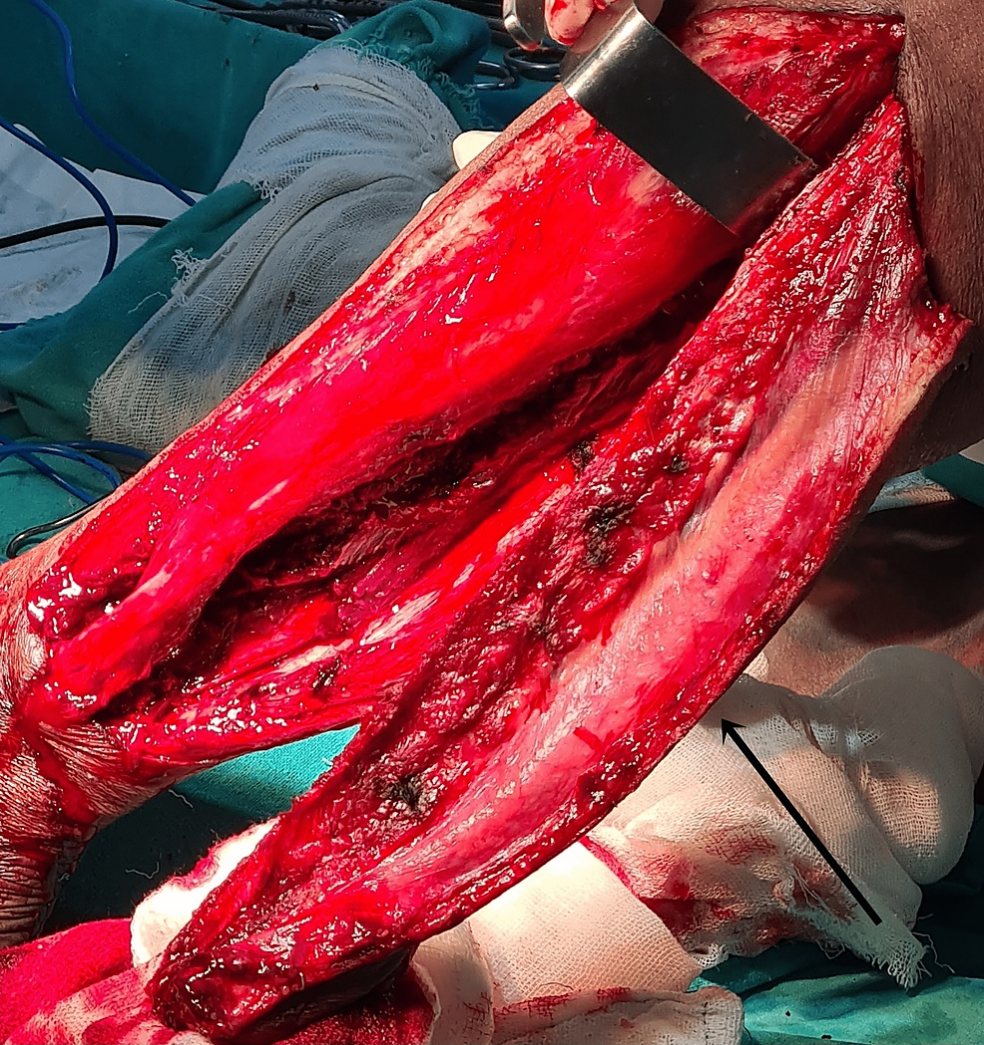
Image showing removal of the fibular free flap from the left lower limb.

**Figure 10:**
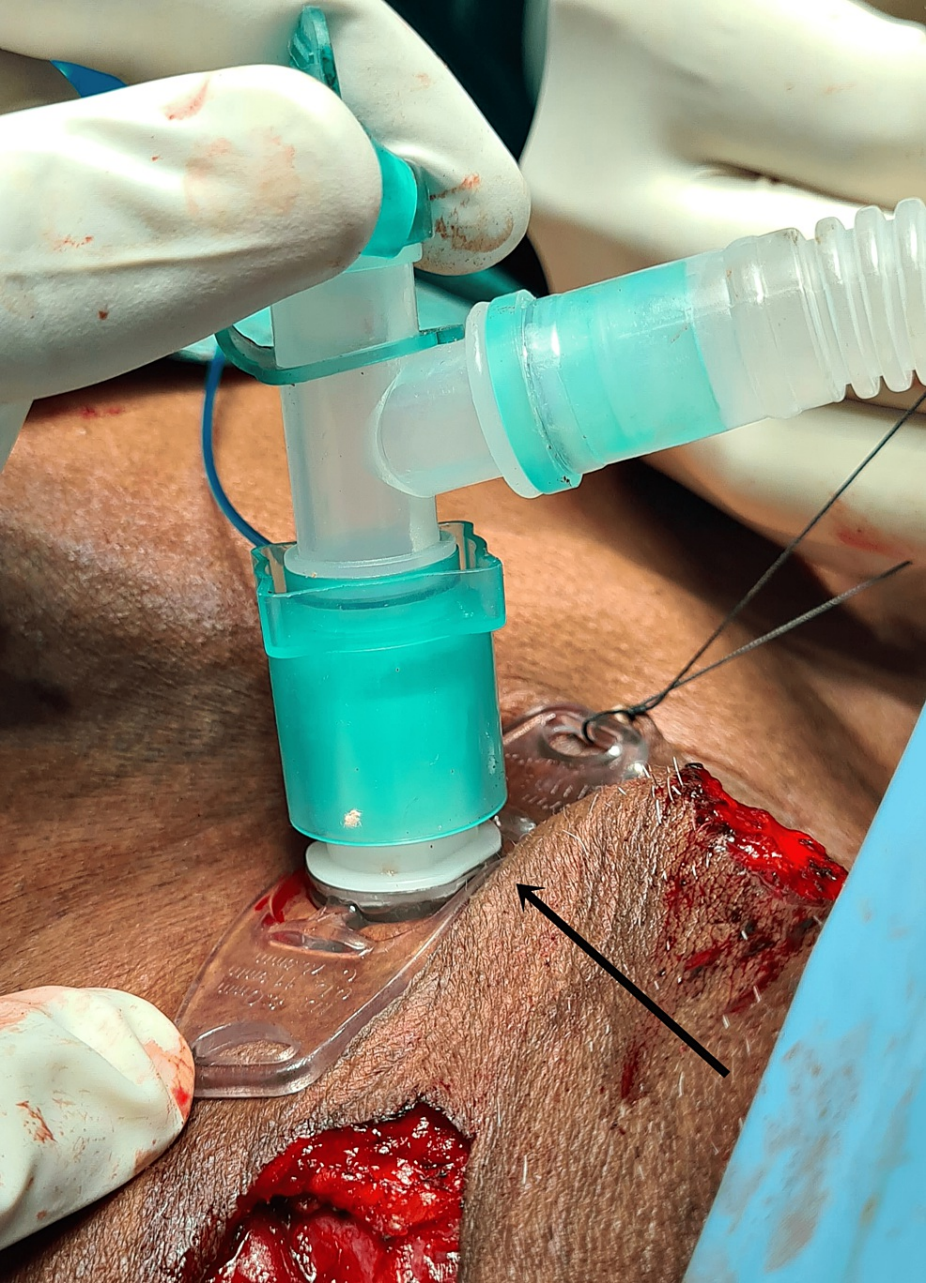
Image showing intraoperative tracheostomy performed with an 8.0 mm cuffed tracheal tube.

After confirmation by direct visualization, five-point auscultation, and end-tidal carbon dioxide capnography, the bronchoscope was removed and propofol 100 mg injection and vecuronium 8 mg injection were given intravenously. The patient was connected to a ventilator with a tidal volume of 450 ml, respiratory rate of 12, peak end-expiratory pressure of 5, and was maintained on 2 L oxygen and 2 L nitrous oxide each along with sevoflurane as an inhalational agent. Throat packing was done on either side of the endotracheal tube intraorally followed by endotracheal tube stitch taken by surgeons after the application of a Vaseline gauze piece to secure the tube and the tube was then fixed at 28 cm at the angle of the nose (Figure 6). Ryles tube was inserted and the patient was handed over to surgeons. Intraoperatively, adequate fluid management and timely replacement of loss were done. The surgery lasted for more than 14 hours. The arterial blood gas (ABG) test was done twice and corrections were given accordingly. A segmental mandibulectomy was performed (Figure 7). Bilateral neck dissection was done to remove the lymph nodes (level I to V) on either side of the neck (Figure 8). A fibular free flap was taken for reconstruction surgery (Figure 9). The frozen sample was then sent for histopathological studies. It was followed by the reconstruction of the mandible and anastomosis. Intraoperative tracheostomy was performed with an 8.0 mm cuffed tracheal tube (Figure 10).

The total blood loss was 1200 ml. The allowable blood loss for the patient was calculated to be 800 ml. A total of 300 ml of packed red blood cells was transfused intraoperatively. Rest replacement was maintained by crystalloids and colloids. Adequate input and output monitoring was done. Post-procedure, the patient was kept sedated with vecuronium and midazolam injection infusion at 5 mg/hour each and the patient was shifted to the ICU and maintained on ventilator support. On postoperative day one, the infusions were stopped, and the patient was gradually weaned off the ventilator and kept on a t-piece on room air after constantly monitoring the peripheral oxygen saturation and breathing pattern. On postoperative day seven, the patient was decannulated and the tracheostomy opening was properly dressed and closed. On postoperative day 12, the patient was discharged without any further postoperative complications.

## Discussion

The management of the airway has developed and gotten increasingly complex in recent years. Problems that are associated with the airways need to be looked at on a frequent basis to find a solution [6]. Cancer is now the biggest cause of mortality in the Asia-Pacific area [7], and it is becoming an increasingly frequent disease overall. Cancers of the head and neck are consistently ranked in the top 10 most common forms of the disease globally [8]. The majority of patients diagnosed with head and neck cancer present with late problems that are connected to the treatment of their airways [9]. Chewing tobacco and gutka, using pan masala, and having a poor diet are the four most prevalent variables that contribute to the development of cancer [8,10]. Oral cancer is a form of head and neck cancer that occurs most often. Both intubation and extubation may be challenging in these cases. This is one of the anaesthetic concerns since it can lead to additional challenges in perioperative airway care. Evaluation, planning, and collaboration with the surgeon are all essential steps in the process of reducing the risk of unneeded complications.

During the perioperative phase, the following variables are responsible for patients with oral cancer having problematic breathing passages [11]: the presence of cancerous development; structural alterations and fibrosis brought on by previous treatment with surgery or radiation; prolonged surgical procedure; a large flap for reconstruction; oedema surrounding the airway as a result of surgical manipulations; the risk of bleeding, primarily due to surgical causes or multiple attempts at airway manipulation; and the possibility of pulmonary aspiration [12]. These are the potential complications that can arise during this type of operation.

Concerns with the anaesthesia include the following: if the patient has a perioral or periglottic growth, bag and mask ventilation may be difficult; laryngoscopes make it possible to easily shatter and dislodge exophytic tumours from their distant locations; they bleed readily, which prevents further glottic visibility; unnoticed tumour expansion to the base of the tongue produces tongue fixation, which in turn creates difficulty with laryngoscopy and intubation; laryngoscopy and glottic visualization might be made more difficult when patients have poor dentition as a consequence of tumour invasion. Video laryngoscopes provide superior views of the airway; nevertheless, they need more room to be introduced and have the potential to dislodge the tumour. A history of radiation only makes the condition worse.

Patients who have previously had radiation and are currently being treated for oral cancer are more likely to need a tracheostomy. In light of these issues, it is necessary to conduct a comprehensive examination and to make preparations for a difficult airway device, which may include an invasive airway approach. Airway management in oral cancer patients is often achieved via the nasal route using general anaesthesia (GA) induction with or without a muscle relaxant [13]. This is due to the fact that the majority of individuals with the disease are recalcitrant. Tracheal intubation is the safest strategy in the majority of situations with expected airway difficulties [14], despite the fact that completing a surgical airway on a conscious patient is sometimes required. Tracheal intubation is performed on conscious patients while they are given topical anaesthesia. The nasal route is favoured over the oral route because it gives surgeons greater space to work with during operations and because patients are able to better tolerate nasal tubes. The American Society of Anesthesiologists (ASA) recommendations detail the essential choices that must be made for the care of all patients [15].

Awake fiberoptic intubation, which is considered the gold standard technique but requires skilled anaesthesiologists [16,17], is one of the preferred techniques. Other preferred techniques include induction with general anaesthesia on a routine basis, with or without a muscle relaxant, and awake fiberoptic intubation. Following the administration of a local anaesthetic spray and intravenous analgesia, an awake laryngoscopy may be conducted. This procedure may be tricky for the patient, as it requires the patient's cooperation and adequate airway blocks by a skilled anaesthesiologist. When using a bag mask for breathing, volatile anaesthetics or/and low-dose propofol are required to be administered. Elective tracheostomies may be performed. The choice is made based on the degree of difficulty in the patient's airway. It has been determined that performing a tracheostomy as the first operation for airway control is not dangerous [18]. The blind nasal method has been linked to an increased likelihood of tumour disruption as well as considerable blood loss. This method is used very seldom due to the availability of the fiberoptic bronchoscope (FOB). In some circumstances, it may also be necessary to perform cricothyrotomy, retromolar intubation, or retrograde intubation. These are all viable options. Retrograde intubations are on the verge of becoming extinct and should only be undertaken by anaesthesiologists who have extensive clinical expertise.

Because repeated attempts may result in hypoxic injury, which can lead to brain damage and death [19], the best technique should be used to secure the airway. All of these techniques are intended to allow for proper preoperative assessment of the airway [20].

## Conclusions

Oral carcinoma cases can pose a great challenge for any anaesthesiologist. Even after proper and thorough preoperative assessment, a trained and experienced anaesthesiologist can come across unexpected airway difficulty owing to the pathology. Hence proper preoperative assessment and adequate intraoperative arrangements need to be done to tackle any such unforeseen difficulty in managing the airway. With the development of technology and science, fibreoptic bronchoscope has become the gold standard for managing such unexpected and difficult airways with anticipated difficult ventilation. In our case, we anticipated facing difficulty and made arrangements for a fibreoptic bronchoscope, which helped in securing the airway with ease and with fewer complications associated with the induction of anaesthesia. Adequate intraoperative haemodynamic management leads to favourable postoperative outcomes and early recovery and discharge from the hospital.
